# Clinical development prospects of siRNA drugs for tumor therapy: analysis of clinical trial registration data from 2004 to 2024

**DOI:** 10.3389/fphar.2025.1637958

**Published:** 2025-09-17

**Authors:** Cai-E. Wang, Delong Zhen, Lukui Yang, Guifang Li

**Affiliations:** The First Affiliated Hospital, and College of Clinical Medicine of Henan University of Science and Technology, Luoyang, China

**Keywords:** siRNA, clinical trials, cancer therapy, drug delivery systems, therapeutic targets

## Abstract

**Background:**

This study systematically compares clinical trial patterns of siRNA drugs in oncology and non-oncology, aiming to inform optimized R&D strategies for oncology.

**Methods:**

Trial phases, sponsor countries, biomarkers, and targets were analyzed for global siRNA trials (2004–2024).

**Results:**

Non-oncology trial dominated (90% of 424 trials), peaking in 2021 (64 trials), and yielded 6 approved drugs for metabolic/genetic diseases. Key non-oncology targets included PCSK9 and HBV. Oncology trials initiated later, primarily focusing on phase I/II studies (60% phase I), targeting solid tumors (40%) and CSF2-related therapies (40%). Clinical trial activity in China commenced in 2019, demonstrating acceleration in 2023, yet overall trial volume remains lower than global benchmarks. Cross-target analysis has pinpointed PTGS2 and TGFB1 as shared targets, indicating the possibility for combination therapy.

**Conclusion:**

Overcoming technical challenges (e.g., targeted delivery) and exploiting multi-target synergies are critical to expanding siRNAs applications in oncology. Success in non-oncology settings demonstrates the translational potential of siRNA technology, however, oncology requires tailored strategies to address complex tumor biology and delivery barriers.

## 1 Introduction

Small interfering RNA (siRNA) comprises double-stranded RNA fragments of 19–23 base pairs. These fragments can be conjugated to carrier systems for tissue-specific delivery, enabling targeted gene silencing in pathogenic tissues. As biomedical research advances, siRNA has emerged as a revolutionary gene therapy approach, garnering significant attention (Hannon, 2002; Castanotto and Rossi, 2009; Dorsett and Tuschl, 2004). Compared to traditional small molecule drugs and antibody-based therapeutics, siRNA drugs offer distinct advantages: shorter research and development cycles, enhanced target specificity, broader therapeutic applicability, and sustained pharmacological effects. These characteristics provide novel strategies and solutions for treating a wide spectrum of challenging diseases, including hereditary disorders, metabolic conditions, and malignant neoplasms (Dorsett and Tuschl, 2004; Alshaer et al., 2021).

In 1998, Andrew Fire and Craig Mello identified the gene-silencing effect of double-stranded RNA in nematode *Caenorhabditis elegans*, designating this phenomenon as RNA interference (RNAi). This discovery established the conceptual foundation for therapeutic RNAi aplications (Fire et al., 1998). The profound significance of this finding was recognized in 2006 when Andrew Fire and Craig Mello were awarded the Nobel Prize in Physiology or Medicine for elucidating the RNAi mechanism, an achievement that accelerated siRNA therapeutic development. As of 31 October 2024, regulatory agencies worldwide have approved six siRNA drugs. These siRNA therapeutics primarily address cardiovascular diseases, cancers, neurological disorders, and immune system-related diseases, targeting key molecules including: transthyretin (TTR), aminolevulinic acid synthase1 (ALAS1), hydroxysteroid (17-beta) dehydrogenase1 (HAO1), proprotein convertase subtilisin/kexin type 9 (PCSK9), and lactate dehydrogenase (LDH).

This study comprehensively analyzes the progress of clinical research on siRNAs, and the results showed that global siRNA drug R&D shows significant field differentiation: non-oncology field dominates (over 90% of clinical projects), with six approved drugs for metabolic/genetic disorders targeting key pathways including PCSK9 and HBV. In contrast, oncology R&D is still at an early stage (60% of phase I), with solid tumors as the main indications (40%), homogeneous targets (CSF2 40%) and a high trial termination rate (28%). (40%), target homogenization (40% for CSF2) and a high trial termination rate of 28%. Studies have further identified common targets in multiple tumors such as PTGS2/TGFB1, which suggesting the potential for combination therapy. Although China’s growth rate has increased in recent years, it still lags behind the international level. Based on the characteristics of siRNA technology, there is a need to breakthrough the bottleneck of targeted delivery and other technologies to expand the application of tumor therapy.

## 2 Methods

**FIGURE 1:**
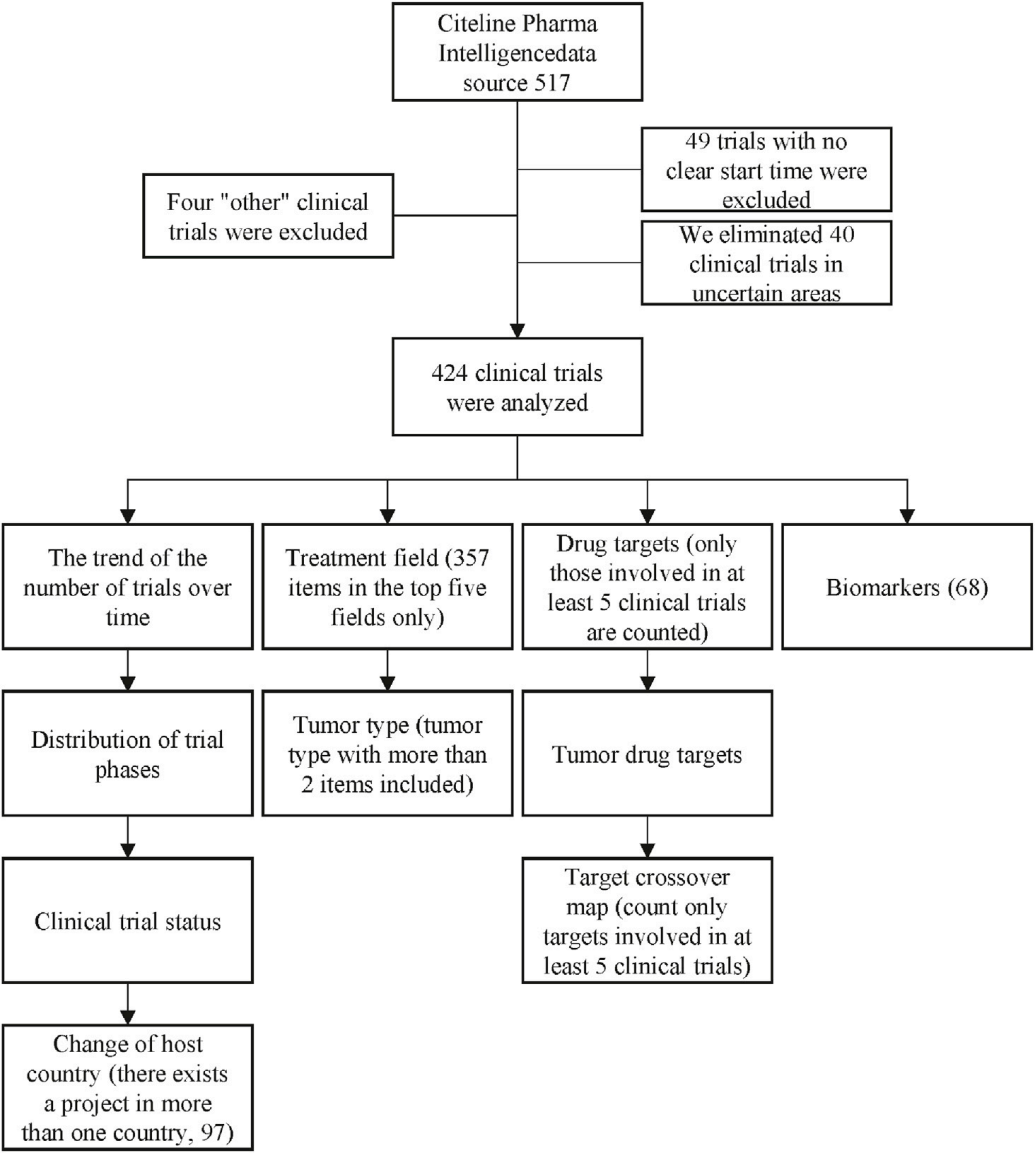
Screening flowchart.

We selected Citeline Pharma Intelligence as our primary data source, which is a comprehensive and up-to-date database of global clinical trial information. To ensure data accuracy, we cross-referenced records with ClinicalTrials.gov and the China Clinical Trials Database. We searched for keywords such as “siRNA”, “RNAi”, and “tumor”, excluding animal studies and non-interventional trials. Meanwhile, we compared trial numbers (NCT/ChiCTR) across multiple databases to ensure consistent information. Two researchers independently reviewed controversial data. From August 2004 to August 2024, a total of 517 siRNA clinical trials were identified. However, 49 trials with unspecified start dates, four trials with “other” research phases, and 40 trials with unspecified regions were excluded from the analysis. Consequently, 424 clinical trials were thoroughly examined. Our analysis encompassed the frequency distribution of trials over time, trial phase distribution, specific indications, drug targets, biomarkers, clinical trial status, changes in sponsoring countries, and characteristics of oncology and non-oncology domains. siRNA drugs have undergone 20 years of development since their inception in 2004, experiencing significant growth until 2016. However, due to the failure of siRNA R&D caused by immature early chemical modification and delivery technologies, the field encountered a setback, resulting in a decrease in clinical trials. Based on this pattern, we divided the timeline into two phases: 2004–2016 and 2017–2024. Using the fisher. test function in R 4.3.2, we performed Fisher’s exact test with Monte Carlo simulation and 20,000 iterations (B = 20,000). All statistical tests were two-sided, with a significance level of α = 0.05. For the indicator-target relationship, we utilized UpSet map to illustrate the distribution and overlap of targets in each indicator. In this map, ‘Set Size’ represents the number of targets, while ‘indicated size’ represents the top five malignancies. The detailed screening process is outlined in Figure 1.

## 3 Results

### 3.1 Temporal trends and field-specific dynamics

An analysis of marketed siRNA drugs revealed a predominance of non-oncology-targeted therapeutics, with six currently approved: inclisiran, vutrisiran, patisiran, lumasiran, givosiran, and nedosiran. These drugs address various conditions, such as familial heterozygous hypercholesterolemia, primary type 1 hyperuricemia, acute hepatic porphyria, and hereditary transthyretin-mediated amyloidosis in adult patients. Detailed information is provided in Table 1.

**TABLE 1:** Summary of marketed non-tumor-oriented siRNA drugs.

Drugs	Company	Trade names	Targets	Approved countries	Approved date	Indications	First approved country
Inclisiran	Novartis	Leqvio	PCSK9	FDA/EMA NMPA/PMDA	2020/12/11	Heterozygous familial hypercholesterolemia	EMA
Vutrisiran	Alnylam	Amvuttra	GalNAc	FDA/EMA/PMDA	2022/6/14	Transthyretin-mediated amyloidosis	FDA
Patisiran	Alnylam	Onpattro	TTR A	FDA/EMA/PMDA	2018/8/10	Hereditary transthyretin-mediated amyloidosis with polyneuropathy	FDA
Lumasiran	Alnylam	Oxlumo	Glycolate oxidase	FDA/EMA	2020/11/23	Primary hyperoxaluria type1	FDA
Givosiran	Alnylam	Givlaari	ALAS1	FDA/EMA/PMDA	2019/11/21	Acute hepatic porphyria	FDA
Nedosiran	Novo Nordisk	Rivfloza	LDHA	FDA	2023/9/29	Primary hyperoxaluria type1	FDA

Concurrently, a broader spectrum of non-oncology-targeted siRNA drugs are undergoing clinical trials for diverse indications including hepatitis B (HBV) and haemophilia. Table 2 provides comprehensive details on these trials (Egli and Manoharan, 2023; Amrite et al., 2023). In contrast, the research and development of oncology-focused siRNA drugs appear to be progressing more slowly, with no applications currently listed for approval.

**TABLE 2:** Summary of non-tumor-directed siRNA drugs in the investigational phase.

Drugs	Company	Drug targets	Indications	Latest research published
ARO-HSD	Arrowhead Pharmaceuticals	HSD17B13	NASH	2022
ACR-520	Arrowhead Pharmaceuticals	cccDNA	CHB	2022
ALN-RSV01	Alynlam	RSV	RSV infection	2024
BMS-986263	BioMimetics Sympathies	HSP47	Advanced hepatic fibrosis	2023
Fitusiran	Sanofi and Alnylam Pharmaceuticals	Antithrombin	Haemophilia	2024
JNJ-73763989	Janssen Pharmaceuticals	GalNAc	HBV	2024
Lepodisiran	Eli Lilly	Lp(a)mRNA	Increased lipoprotein A	2024
Olpasiran	Amgen	Lp(a)mRNA	Increased lipoprotein A	2024
Zerlasiran (SLN360)	Silence Therapeutics	Apolipoprotein	Increased lipoprotein A	2024
SLN124	Silence Therapeutics	GalNAc	Hereditary Haemochromatosis Type1	2023
Siran-027	Siran	VEGFR-1	Choroidal neovascularization	2010
Teprasiran	Quark Pharmaceuticals	p53	Acute kidney injury in high-risk patients undergoing cardiac suroerv	2021
TRK-250	Toray Industries	TGF-β1	Idiopathic Pulmonary Fibrosis	2023
Plozasiran	Arrowhead Pharmaceuticals	APOC3	Hypertriglyceridemia	2024
PF-04523655	Quark Pharmaceuticals	RTP801	Diabetic Macular Edema	2012

**FIGURE 2:**
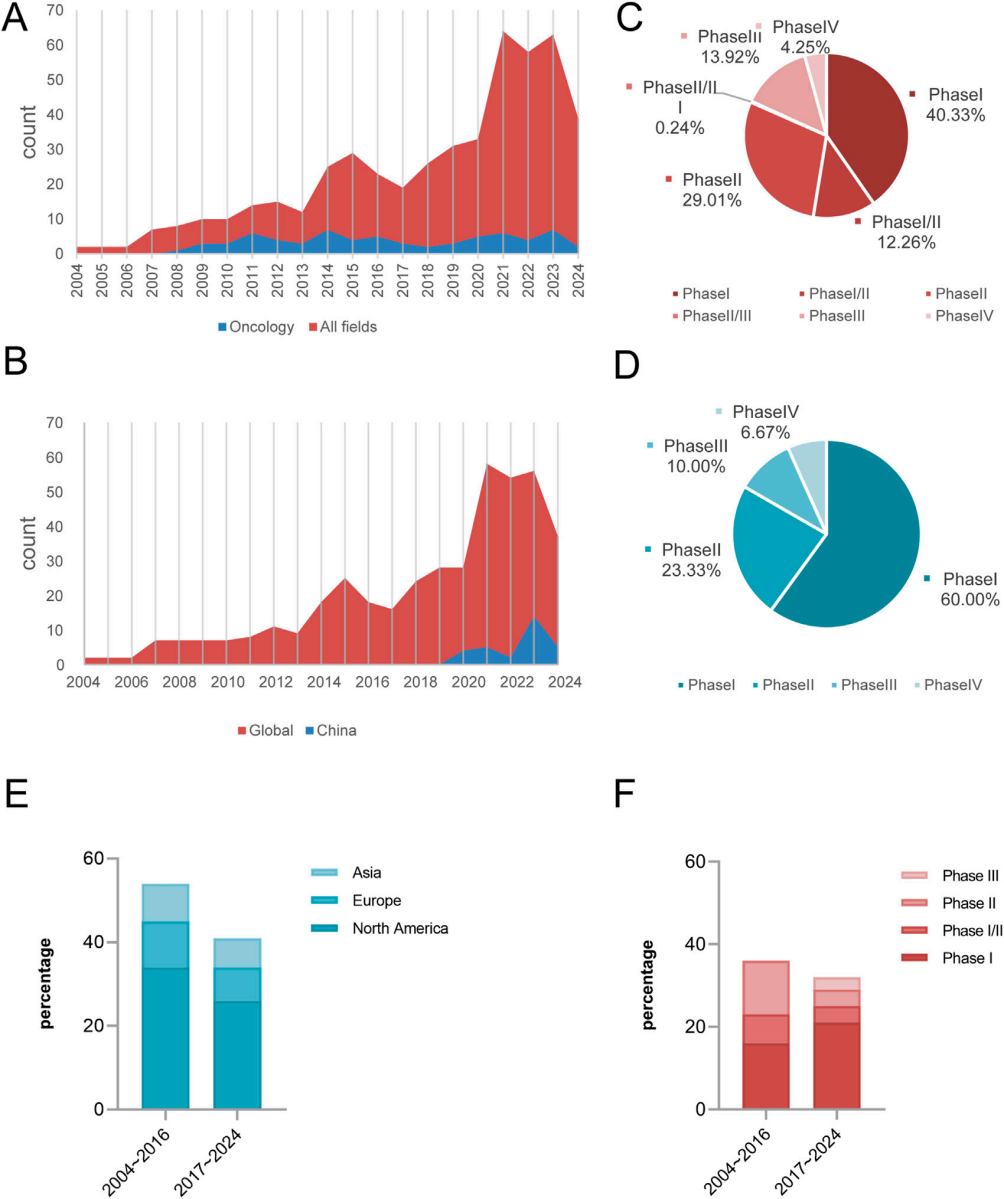
Highlights temporal/regional trends, phase disparities, and oncology vs. non-oncology dynamics. Global trends in siRNA clinical trials (2004–2024): Field-specific growth **(A,B)**, Distribution of all areas during the trial phase **(C)**. Distribution of oncology clinical trials across different phase **(D)**. The evolution of regional distribution of global siRNA clinical trials for oncology (2004∼2016 vs. 2017∼2024, *P* = 0.994) **(E)**. Phased changes in the composition of global siRNA oncology clinical trials (2004∼2016 vs. 2017∼2024, *P* = 0.024) **(F)**.

We analysed 424 clinical trials of siRNA drugs initiated worldwide between 2004 and 2024. Overall, the number of trials demonstrated an upward trend, accelerating markedly after 2013 and peaking in 2021 (64). Among these oncology-targeted siRNA drugs development commenced later with fewer aggregate trails, it exhibited parallel growth patterns with notable peaks in 2014 and 2023. The development trajectory closely mirrors that of all siRNA drugs, with minor fluctuations. In comparison with the clinical trials of oncology-targeted siRNA drugs, the number of trial projects for siRNA drugs in other fields is notably higher, accounting for 90.63% of the total clinical trials in 2021 (Figure 2A).

Since the start of clinical trials of siRNA drugs in China in 2019, the number of projects will remain at a low level until 2022, and the number of related trials started to show an increasing trend in 2023. Although the research in this field started later than the international advanced level, the existing project size is limited and there is a gap in the overall development, the research process has always maintained a stable upward trend (Figure 2B).

From the characteristics analysis of the distribution of clinical trial stages, phase I and II trials dominate both oncology and non-oncology fields, with phase I clinical trials in oncology being particularly prominent, accounting for 60% of the total. Due to the advantages of a larger project base and earlier implementation time, the clinical research design of non-oncology has the characteristics of a refined exploration of transitional phases such as phase I/II and phase II/III. In contrast, oncology clinical trials are at an early stage of development and the research staging model has not yet formed a complete system at this stage (Figures 2C,D).

This study analyzed two time periods: 2004–2016 and 2017–2024. In terms of individual sponsoring countries, North America conducted more clinical trial projects than Europe and Asia during both time periods. From 2004 to 2016 to 2017–2024, the number of oncology clinical trial projects decreased across regions, though the distribution ratio of trial numbers across regions did not change significantly (*P* > 0.05). However, there were significant differences in the distribution of clinical trial phases between the two time periods (*P* = 0.024), manifested by the emerged of Phase III trials, accounting for 9.4% (3/32), whereas no such trials were observed in the first period; Phase II trials decreased by 23.6 percentage points (36.1% → 12.5%); and the proportion of Phase I trials increased by 21.2 percentage points (44.4% → 65.6%) (Figures 2E,F).

### 3.2 Biomarker prioritization and field-specific target landscapes

**FIGURE 3:**
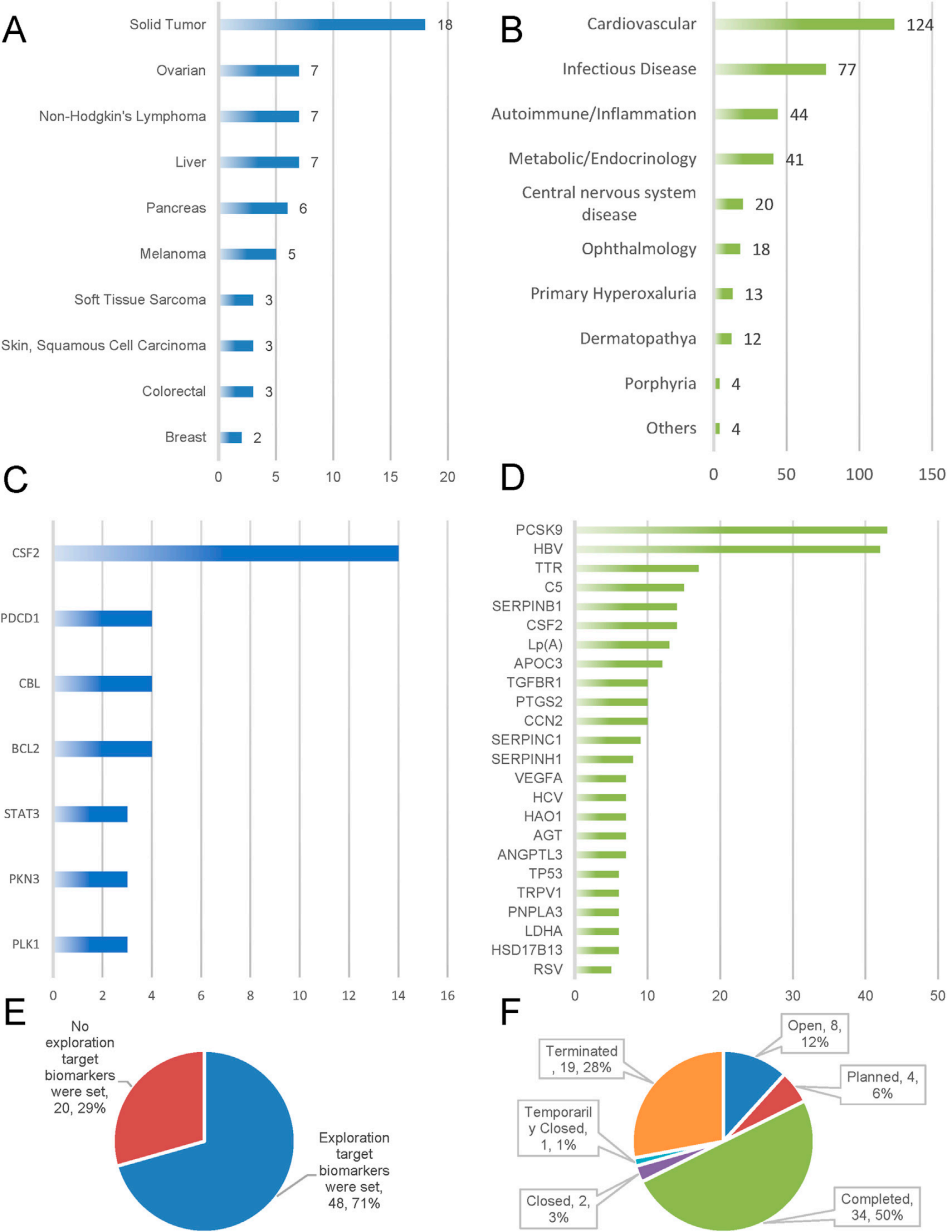
Emphasizes biomarker adoption, trial outcomes, and target/indication clustering. Indications and targets **(A–D)**; Biomarker prioritization **(E)**; Trial status in oncology siRNA clinical development **(F)**.

Figure 3E illustrates that approximately 71% (60/102) of the tumor-targeted siRNA clinical trials focused on examining biomarkers. Among the global clinical trials for tumor siRNA, 12% are currently ongoing, 50% have been completed, 6% are in the planning stage, 1% were temporarily suspended, 3% have been closed, and 28% have been terminated (Figure 3F).

We analyzed the reasons for trial termination in the oncology field (n = 19). Business decisions accounted for the majority of cases (36.8%), followed by trials planned but never initiated (21.1%), lack of efficacy (15.8%), safety/adverse effects (10.5%), and poor enrollment rates (5.3%). Analysis of trial outcomes (n = 34) revealed primary endpoint achievement in 47.1% of completed trials. Unknown and indeterminate outcomes accounted for 50.0% and 2.9% of cases respectively, with no observed instances of primary endpoint failure. See Table 3.

**TABLE 3:** Reasons for termination and outcome distribution of clinical trials of siRNA drugs for tumors.

Reasons for trial termination (n = 19)	Percentage (frequency)
Business decision (Pipeline reprioritization; Drug strategy shift)	36.8% (7)
Planned but never initiated	21.1% (4)
Lack of efficacy	15.8% (3)
Safety/adverse effects	10.5% (2)
Unknown	10.5% (2)
Poor enrollment	5.3% (1)
Trial outcome (n = 34)	Percentage (frequency)
Outcome unknown	50.0% (17)
Positive outcome/primary endpoints met	47.1% (16)
Outcome indeterminate	2.9% (1)
Negative outcome/primary endpoints not met	0%

Therapeutic area analysis revealed cardiovascular, infectious, autoimmune, endocrine and central nervous system diseases as the top five indications for siRNA clinical trial. This trend is evidenced by approved and pipeline agents (e.g., inclisiran, BMS-986263, lepodisiran, etc.) primarily targeting infectious diseases (e.g., hepatitis B) and metabolic disease (e.g., diabetes) in Tables 1, 2. Breakthroughs in these areas may be attributed to the maturation of liver-targeted delivery systems (e.g., GalNAc coupling technology), which have made metabolic disorders (e.g., PCSK9 targeting) more amenable to efficient gene silencing. R&D breakthroughs in specific indications often create a demonstration effect, leading to a surge of research in that area, which in turn drives technology expansion into other therapeutic areas. This explains why clinical exploration of siRNA therapeutics in oncology has lagged behind other therapeutic categories.

An integrated analysis of Tables 1, 2 suggests that the success of marketed non-oncology siRNA drugs can be attributed to clear target-action mechanisms, precise indication targeting (focused on rare diseases such as hereditary amyloidosis and primary hyperoxaluria), and effective support of quantifiable surrogate endpoints. These factors facilitate efficient clinical translation pathways. The pipeline under development further reinforces the advantage of liver targeting and expands indications to chronic disease spectrums. Meanwhile, the R&D landscape is shifting from Alnylam’s dominance to collaborative participation by multiple companies. In contrast, the tumor siRNA field faces significant challenges, including target fragmentation (e.g., CSF2 accounts for 40%, yet has limited efficacy, and the remaining targets are distributed in a fragmented manner), inadequate endpoint assessment objectivity (e.g., lack of reliable biomarkers), and a vicious cycle in R&D timelines (e.g., target validation gaps, 28% high termination rate, and Phase I stagnation). Breakthroughs in non-oncology fields have provided concrete optimization pathways for overcoming challenges in oncology R&D.

Our analysis of clinical trials for oncology-targeted siRNA drugs revealed that solid tumors constituted the largest proportion of indications, followed by ovarian cancer and non-Hodgkin’s lymphoma. This finding aligns with the information presented in Figures 2C,D, which indicates that clinical trials are primarily concentrated in phases I and II. These early-phase trials primarily focus on pharmacokinetics and preliminary pharmacodynamics as their endpoints, explaining the predominance of solid tumor indications. Furthermore, ongoing clinical trials are exploring applications in liver cancer, pancreatic cancer, and melanoma (Figure 3A).

Analysis of siRNA drug frequency by target revealed that in the non-oncology domain, the three most prevalent targets were PCSK9 (14.98%), HBV (14.63%), and TTR (5.92%). In oncology projects, the top three targets were colony stimulating factor 2 (CSF2) (40%), programmed cell death 1 (PDCD1) (11.43%), and Cbl protooncogene B (CBL) (11.43%) (Figures 3C,D).

### 3.3 Intertumoral target heterogeneity and common pathways across cancers

**FIGURE 4:**
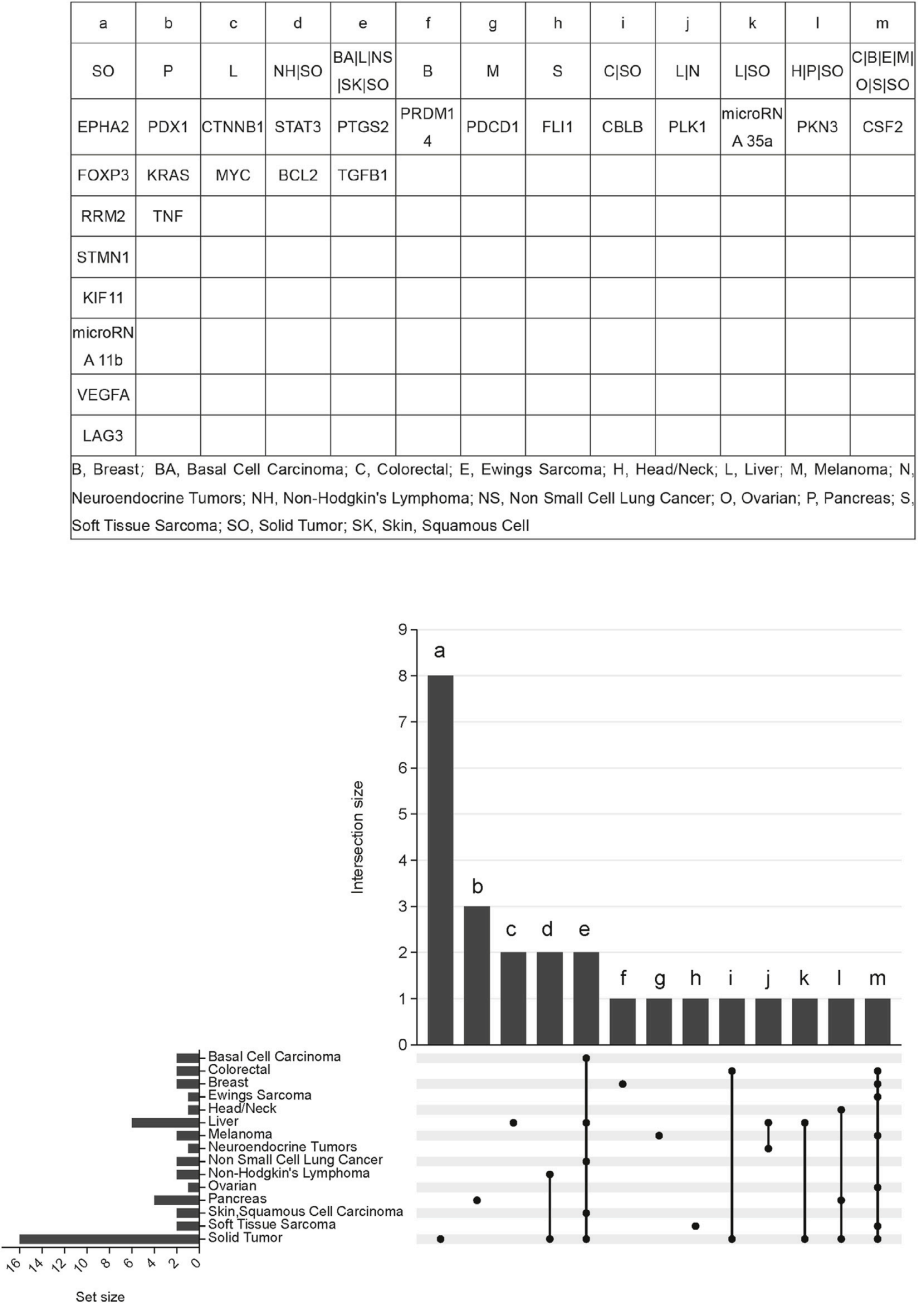
Indication-target relationship of siRNA therapeutics clinical trials for tumors.

Tumors can harbour multiple distinct molecular targets, and it is common for different tumor types to share certain targets. Solid tumors exhibit multiple targets, including EPH receptor A2 (EPHA2), forkhead box P3 (FOXP3), ribonucleotide reductase regulatory subunit M2 (RRM2), stathmin 1 (STMN1), kinesin family member 11 (KIF11), and microRNA 11b. Pancreatic tumors specifically feature three targets: pancreatic and duodenal homeobox 1 (PDX1), KRAS protooncogene GTPase (KRAS), and tumor necrosis factor (TNF). Several cancer types, including basal cell carcinoma, hepatocellular carcinoma, non-small cell lung cancer, cutaneous squamous cell carcinoma, and solid tumors, share two common targets: prostaglandin-endoperoxide synthase 2 (PTGS2) and transforming growth factor beta 1 (TGFB1). Additionally, CSF2 serves as a shared target among colorectal cancer, breast cancer, Ewing’s sarcoma, melanoma, ovarian cancer, soft tissue sarcoma, and solid tumors. Non-Hodgkin’s lymphoma and solid tumors share two targets: signal transducer and activator of transcription 3 (STAT3) and BCL2 apoptosis regulator (BCL2). A detailed overview is shown in Figure 4.

## 4 Discussion

Between 2004 and 2016 and 2017∼2024, the number of siRNA drug clinical trial projects increased from 123 to 301. The total number of projects in the latter eight-years period was 2.4 times greater than in the initial twelve-year span. This rise indicates that siRNA therapeutics has become an increasingly prominent area of development. Since 2016, researchers have employed advanced chemical modifications and targeted delivery systems to address the challenges of siRNA instability and susceptibility to degradation by RNA enzymes *in vivo*. These advancements have markedly improved siRNA half-life and enhanced drug penetration into cells and tissues (Parmar et al., 2016; Dong et al., 2019; Mainini and Eccles, 2020; Jorge et al., 2020; Gupta et al., 2021; Khvorova and Watts, 2017). However, tumor-targeted siRNA drugs did not exhibit a similar growth trajectory post-2016. This discrepancy may be attributed to the unique physiological characteristics of tumor tissues, which present additional challenges for drug development and launch. In tumor environments, siRNA nanoparticles must accumulate in the target tissue and penetrate deeply to effectively silence target genes. Research has shown that the importance of tumor penetration is often underestimated (Wu et al., 2016; Guo et al., 2024). Biopsy samples from different regions of the same tumor have demonstrated significant variations in the gene-silencing efficacy of siRNA drugs (MacEwan et al., 2010). Previously, efforts to enhance siRNA drug retention and penetration emphasized the enhanced permeability and retention (EPR) effect, primarily based on differences in vascular structure and permeability between tumor and normal tissues. However, due to the heterogeneous vascular characteristics of different tumor types and growth stages, siRNA nanoparticles exhibit varied biological distribution patterns across tumor types and stages (Wang et al., 2017), presenting new challenges for siRNA oncology drug development. Furthermore, tumor siRNA drug development often necessitates screening multiple candidate target genes and elucidating their roles in healthy tissues to mitigate potential toxic side effects (Kara et al., 2022; Trajanoska et al., 2023; Liu et al., 2023). Additionally, reliable biomarkers are crucial for assessing clinical responses to siRNA treatment (Kara et al., 2022; Wu et al., 2022). Consequently, despite the increasing number of siRNA drugs being developed in other therapeutic areas since 2016, progress in oncology therapy remains limited. Simultaneously, the R&D cluster effect of marketed drugs warrants attention. Non-oncology siRNA therapeutics, represented by Inclisiran (Ray et al., 2020) and Vutrisiran (Adams et al., 2023), have formed a significant driving effect in their respective therapeutic areas, while tumor siRNA drugs, which have not yet been clinically translated, lack such a demonstration effect and face the technical difficulties mentioned above. Nevertheless, these challenges have gradually gained attention from pharmaceutical researchers. It is anticipated that through collaborative efforts, these obstacles will be progressively overcome, potentially leading to a significant advancement in tumor-targeted siRNA drug development.

From 2017 to 2024, there was a structural shift in tumor siRNA clinical trials (*P* = 0.024). The proportion of Phase I trials increased by 21.2 percentage points (from 44.4% to 65.6%), reflecting the industry’s adoption of a decentralized exploratory strategy. This strategy breaks down traditional Phase II trials into small-scale Phase I trials that target specific areas. This is an attempt to address the 28% risk of termination. Phase II trials saw a collapse of 23 percentage points.6 percentage points (36.1%–12.5%), revealing deficiencies in delivery efficiency. Most projects failed to meet efficacy validation thresholds. Phase III trials accounted for 9.4% of trials, marking the entry of first-generation candidate drugs into the confirmatory phase. However, this figure remains significantly below the industry average of 30% in non-oncology fields, exposing the fragility of the R&D pipeline. This pattern of early expansion and late scarcity highlights the dual challenges of high attrition and low conversion rates in tumor siRNA R&D. Compared to the success of six drugs launched in non-tumor fields, the declining trend in the total number of tumor trials from 2017 to 2024 further reflects the cautious attitude of capital toward conversion prospects.

This study reveals three major challenges facing the development of tumor-specific siRNA through an in-depth analysis of the reasons for and outcomes of clinical trials involving it. First, capital-sensitive advancement mechanisms: 36.8% of trials were terminated due to adjustments in commercial strategies, reflecting capital’s cautious assessment of the potential for translation, especially compared to the six drugs already on the market in non-tumor fields (This is consistent with our inference about the contents of Figures 2E,F; Table 1). Second, technical validation pressure: The 21.1% of trials that were not initiated and the 15.8% of trials with insufficient efficacy point to barriers to feasibility and bottlenecks in delivery efficiency in early-stage development. Disrupted Clinical Evidence Chain: The high rate of unknown outcomes (50.0%) exposes the fragility of follow-up systems and data disclosure gaps. The statistical illusion of a 0% primary endpoint non-achievement rate stems from the early elimination of ineffective projects (e.g., projects with insufficient efficacy that do not enter endpoint analysis). The reliability of the 47.1% primary endpoint achievement rate is disrupted by the high proportion of unknown outcomes (50.0%) and result uncertainty (2.9%), which requires further data validation. Furthermore, even projects that achieve the primary endpoint must navigate multiple efficacy validation hurdles in Phase II/III trials due to the high proportion of Phase I projects in the oncology field (Figure 2D). In summary, these findings highlight core contradictions in oncology siRNA R&D, including the risk of a broken clinical evidence chain (conflict between long R&D cycles and patient survival periods), inefficient delivery systems, and capital-sensitive advancement mechanisms. While capital caution is prevalent across the drug development field, it is significantly amplified in tumor siRNA due to the high termination rate (28%). The risk of a broken clinical evidence chain necessitates optimizing trial design from the beginning, such as by prioritizing localized lesions. Skin cancer indications, for example, can shorten the development timeline. The inefficiency of drug delivery systems requires breakthroughs in delivery technology.

This early-stage predominance—particularly acute in oncology where only 9.4% of trials reach Phase III—demands a comprehensive approach during siRNA drug design, emphasizing optimal delivery strategies and a thorough understanding of pharmacokinetics, pharmacodynamics, and active metabolites. Compared to conventional drugs, small nucleic acid drugs face efficiency challenges due to the need to traverse the cytosol membrane to target mRNA in the cytoplasm or nucleus (Zhang et al., 2021). Their instability, larger molecular structure, and negative charge make them susceptible to nuclease degradation and renal clearance, with unmodified siRNA having a blood half-life of only 5 min (Gao et al., 2009; Schenk et al., 2008; Godinho et al., 2022; Biscans et al., 2019). Consequently, effective delivery strategies represent a major obstacle for siRNA clinical translation. Initially, siRNA drug therapies were primarily confined to localized treatments, such as intravitreal injections (Rajappa et al., 2010). The delivery of small interfering RNA (siRNA) to target tissues or cell types is influenced by various factors, including the administration route, biological barriers, tissue or cell uptake, and escape from the endosome. Without a delivery conjugate, completely chemically stable siRNA is essentially ineffective. Therefore, an appropriate drug delivery system is crucial for the success of siRNA drug development. Current siRNA delivery systems, classified by carrier type, primarily include lipid nanoparticles (LNPs), exosomes, polymer nanoparticles, and inorganic nanoparticles (Tang and Khvorova, 2024). Of the six currently available global siRNA drugs, five use LNP (e.g., patisiran) or its conjugation with other technologies. In Phase III clinical trials, more than 70% of drugs use LNP or other technologies. Excluding Exosomes entering Phase I clinical trials (e.g., ER2001), the remaining two are still in the early stages of clinical research (Setten et al., 2019; Adams et al., 2018). In contemporary systemic therapies, siRNA drugs are typically delivered using nanoparticle carrier systems, encapsulated by lipids or polymers to enhance cellular uptake, intracellular processing, and targeting to subcellular sites of action (K et al., 2019). Among these, lipid nanoparticle (LNP) delivery systems show the greatest promise. Several clinical trials of siRNA delivery using LNP formulations have been completed, exemplified by the FDA-approved siRNA-based LNP therapeutic patisiran (K et al., 2019). In oncology, LNP-based siRNA drugs primarily focus on treating solid tumors, including hepatocellular carcinoma, prostate cancer, and pancreatic cancer, with some trials targeting other solid tumors such as ovarian cancer, breast cancer, and glioma (El Moukhtari et al., 2023). Despite slow translation rates, numerous candidates remain in clinical trials for solid tumor treatment. However, LNP has certain limitations: intravenous administration can cause adverse reactions and significant liver accumulation, and it is not suitable for disseminated or metastatic tumors. Furthermore, the diverse tumor microenvironment presents substantial barriers to LNP penetration, including vascular abnormalities, hypoxia, and acidic environments (El Moukhtari et al., 2023). Despite LNP’s excellent performance in liver targeting, its penetration efficiency in solid tumors such as pancreatic cancer and brain tumors remains to be optimized. Nonetheless, LNP remains the preferred carrier class for siRNA molecules due to its simple preparation process and favourable safety profile. Future studies should comprehensively evaluate LNP’s potential, including interactions with the tumor microenvironment and its combination with other drugs.

As a cutting-edge strategy for tumor-targeted therapies, siRNA drugs show significant development potential in the field of tumor microenvironment regulation. Research data indicate that CSF2 (granulocyte-macrophage colony-stimulating factor, GM-CSF) has emerged as the most actively pursued target for siRNA drug development (Figure 2F). This cytokine is secreted by stromal cells such as T cells and macrophages (Becher et al., 2016) and drives tumor progression through a dual mechanism: first, by inducing the polarization of tumor-associated macrophages (TAMs) toward an immunosuppressive M2 phenotype and promoting the secretion of immunosuppressive factors such as IL-10 and TGF-β (Li et al., 2020; Greter et al., 2012); second, by activating the MAPK signaling pathway to accelerate tumor cell proliferation and migration (Li et al., 2020). Among them, CSF2-mediated TAM reprogramming is a key regulatory node affecting tumor growth and metastasis (Ji et al., 2023). At the clinical translational level, Vigil, a CSF2-targeted siRNA drug developed by Gradalis, has demonstrated breakthrough activity. A Phase II study published in Clinical Cancer Research 2023 confirmed that Vigil in combination with temozolomide/irinotecan regimen resulted in disease control in 60% of patients with recurrent Ewing’s sarcoma and that efficacy showed a significant correlation with the dynamics of circulating tumor DNA (Anderson et al., 2023). This regimen has a favorable safety profile and extends the positive efficacy signals observed in earlier Phase I (19 solid tumors) and Phase IIa/b (ovarian cancer) studies. It therefore provides a promising paradigm for solid tumor immunotherapy.

Activation of IL-6/STAT3, a classic signaling pathway in tumor cells, begins when IL-6 binds to IL-6Rα and gp130 receptor subunits on the membrane surface to form a complex that triggers phosphorylation of JAK kinase and ultimately activation of STAT3 (Hirano, 2021). In addition to IL-6, growth factors such as FGF, IGF, and EGF mediate STAT3 phosphorylation via cognate receptors (Hillmer et al., 2016). Activated STAT3 drives tumor progression by regulating the expression of genes involved in survival, proliferation, angiogenesis, and immune escape (Zou et al., 2020). Consequently, STAT3 inhibitors represent potential therapeutic targets (Mohan et al., 2022). Reflecting this, Novo Nordisk developed DCR-STAT3 to initiate a Phase I clinical trial (NCT06098651) in August 2023 to evaluate the safety, tolerability and pharmacokinetic profile of this siRNA drug in patients with refractory solid tumors.

The RING-type E3 ubiquitin ligase activity of Cbl-b, an important member of the Cbl junction protein family, makes it a key negative regulator of lymphocyte and natural killer cell (NK cell) activation (Augustin et al., 2023). Functional inhibition of this protein significantly increases the activation threshold of immune cells, a breakthrough discovery that provides a theoretical basis for the development of novel immune checkpoint modulators. Based on the above mechanism, APN401, developed by invIOs GmbH, innovatively uses *in vitro* treatment of autologous peripheral blood mononuclear cells (PBMC) to remodel cellular immune function through a siRNA-mediated Cbl-b transient silencing strategy. Recently published data from the Phase I clinical trial of APN401 in solid tumors showed that of the subjects who completed the full course of intravenous infusion, four patients (two with pancreatic cancer and one each with colon and kidney cancer) achieved disease stabilization during the treatment cycle and no dose-limiting toxicity events were observed in any of the cases, confirming that the therapy has a manageable safety profile (NCT03087591).

Notably, STP707/STP705, the core product of the pipeline developed by SUNON PHARMACEUTICAL, adopts a dual-target silencing strategy. By concurrently silencing TGF-β1 and COX-2 gene expression, it achieves synergistic multi-pathway regulation. At present, STP707 has been approved by the U.S. FDA for IND, and the approved indications cover the three major areas of cholangiocarcinoma, non-melanoma skin tumors and pathological scarring. The related multi-center clinical trials are currently advancing (NCT05037149).

## 5 Conclusion

This study statistically analyzed 424 siRNA drug clinical trials, focusing on oncology therapeutics across trial volume, indications, targets, and status. Analysis reveals oncology siRNA drugs remain in early-stage R&D, with limited trial numbers and development constrained by tumor penetration barriers and delivery challenges. Nevertheless, siRNA drugs constitute an essential frontier in cancer therapy due to abbreviated development cycles and precise targeting. We propose this work as a strategic reference for optimizing siRNA-tumor adaptive drug design, overcoming target innovation deficits (e.g., CSF2 homogeneity) and delivery limitations, thereby accelerating clinical translation of this drug class.

## Data Availability

The data analyzed in this study is subject to the following licenses/restrictions: none. Requests to access these datasets should be directed to zhendelong0@163.com.
